# 2,9-Bis(5-sulfanylidene-4,5-di­hydro-1,3,4-oxa­diazol-2-yl)-1,10-phenanthroline dimethyl sulfoxide disolvate

**DOI:** 10.1107/S1600536814003304

**Published:** 2014-02-22

**Authors:** Md. A Rahman, Mohammad Karim, Md. Arifuzzaman, Tasneem Siddiquee, Lee M. Daniels

**Affiliations:** a Department of Chemistry, Boswell Science Complex, Tennessee State University, Nashville, 3500 John A. Merritt Blvd, Nashville, TN 37209, USA; b Department of Chemistry, Iowa State University, Ames, IA 50011-3111, USA; c Agilent Technologies, 5301 Stevens Creek Blvd, Santa Clara, CA 95051, USA

## Abstract

In the title compound, C_16_H_8_N_6_O_2_S_2_·2C_2_H_6_OS, the phenanthroline mol­ecule resides on a twofold axis, and the asymmetric unit also contains a slightly disordered [occupancy ratio for S atom of 0.95 (3):0.047 (3)] mol­ecule of dimethyl sulfoxide. The O atoms of the solvent mol­ecule accept hydrogen bonds from the N—H groups of the five-membered 2,3-di­hydro-1,3,4-oxa­diazole-2-thione ring. This ring is nearly coplanar with the phenanthroline ring, with a dihedral angle between their least-squares planes of 8.86 (6)°. In the crystal, the mol­ecules are linked by C—H⋯O inter­actions.

## Related literature   

For the biological activity of the oxa­diazole unit, see: Chen *et al.* (2000 ▶); Sun *et al.* (2013 ▶); El-Emam *et al.* (2004 ▶). For their anti­cancer activity, see: Zhang *et al.* (2011 ▶); Gudipati *et al.* (2011 ▶); Abou-Seri (2010 ▶). For related structures, see: Saeed *et al.* (2010 ▶); Fun *et al.* (2011 ▶); El-Emam *et al.* (2012 ▶, 2013 ▶). 
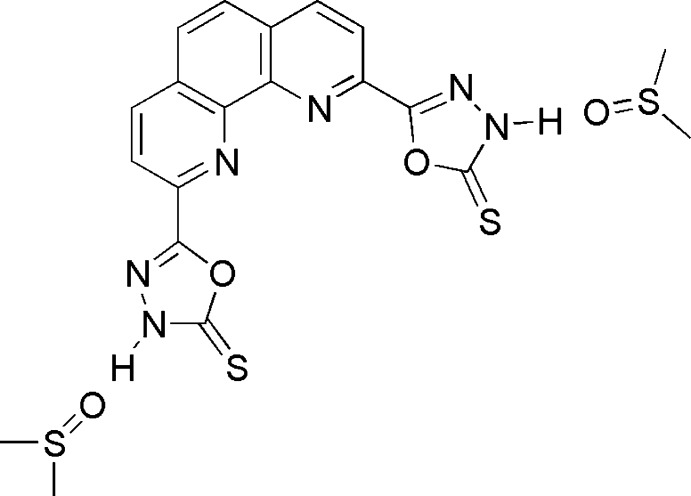


## Experimental   

### 

#### Crystal data  

C_16_H_8_N_6_O_2_S_2_·2C_2_H_6_OS
*M* *_r_* = 536.66
Monoclinic, 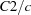

*a* = 14.113 (11) Å
*b* = 11.161 (8) Å
*c* = 16.708 (12) Å
β = 112.837 (14)°
*V* = 2425 (3) Å^3^
*Z* = 4
Mo *K*α radiation
μ = 0.43 mm^−1^
*T* = 277 K
0.42 × 0.26 × 0.15 mm

#### Data collection  

Rigaku XtaLAB mini diffractometer
Absorption correction: multi-scan (*CrystalClear*; Pflugrath, 1999 ▶) *T*_min_ = 0.840, *T*_max_ = 0.938
5592 measured reflections
2741 independent reflections
1691 reflections with *I* > 2σ(*I*)
*R*_int_ = 0.039

#### Refinement  

*R*[*F* ^2^ > 2σ(*F* ^2^)] = 0.067
*wR*(*F* ^2^) = 0.171
*S* = 1.03
2741 reflections
164 parameters
H atoms treated by a mixture of independent and constrained refinement
Δρ_max_ = 0.30 e Å^−3^
Δρ_min_ = −0.59 e Å^−3^

### 

Data collection: *CrystalClear* (Pflugrath, 1999 ▶); cell refinement: *CrystalClear*; data reduction: *CrystalClear*; program(s) used to solve structure: *SHELXS97* (Sheldrick, 2008 ▶); program(s) used to refine structure: *SHELXL97* (Sheldrick, 2008 ▶); molecular graphics: *ORTEP-3 for Windows* (Farrugia, 2012 ▶); software used to prepare material for publication: *WinGX* (Farrugia, 2012 ▶) and *publCIF* (Westrip, 2010 ▶).

## Supplementary Material

Crystal structure: contains datablock(s) global, I. DOI: 10.1107/S1600536814003304/fj2661sup1.cif

Structure factors: contains datablock(s) I. DOI: 10.1107/S1600536814003304/fj2661Isup2.hkl

Supporting information file. DOI: 10.1107/S1600536814003304/fj2661Isup3.cml

CCDC reference: 950902

Additional supporting information:  crystallographic information; 3D view; checkCIF report

## Figures and Tables

**Table 1:** Hydrogen-bond geometry (Å, °)

*D*—H⋯*A*	*D*—H	H⋯*A*	*D*⋯*A*	*D*—H⋯*A*
N2—H2⋯O2	0.89 (4)	1.73 (4)	2.617 (4)	172 (4)
C9—H9*C*⋯O1^i^	0.96	2.62	3.399 (7)	138
C10—H10*B*⋯O2^ii^	0.96	2.57	3.317 (6)	135
N2—H2⋯S2*B*	0.89 (4)	2.36 (5)	3.10 (3)	140 (3)

Symmetry codes: (i) 
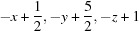
; (ii) 
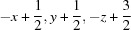
.

**Fig. 1.**
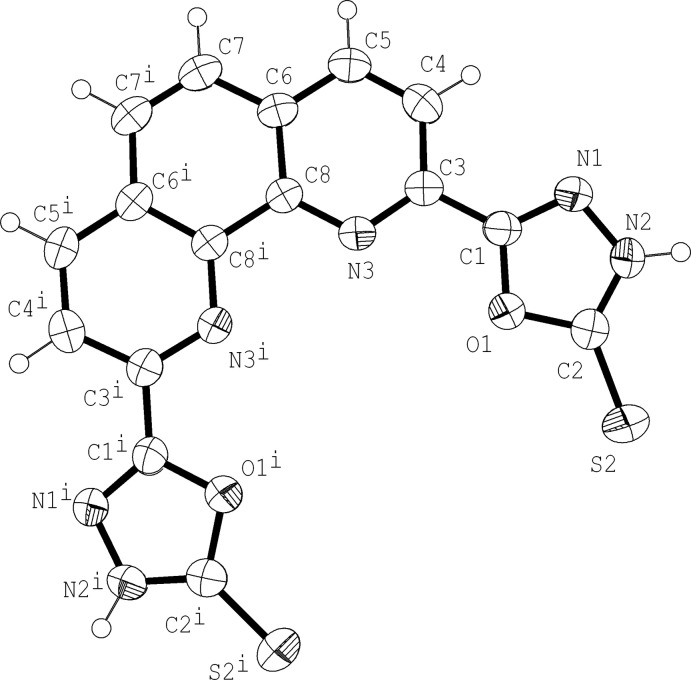
Molecular structure showing 50% probability displacement ellipsoids. DMSO molecules are not shown

**Fig. 2.**
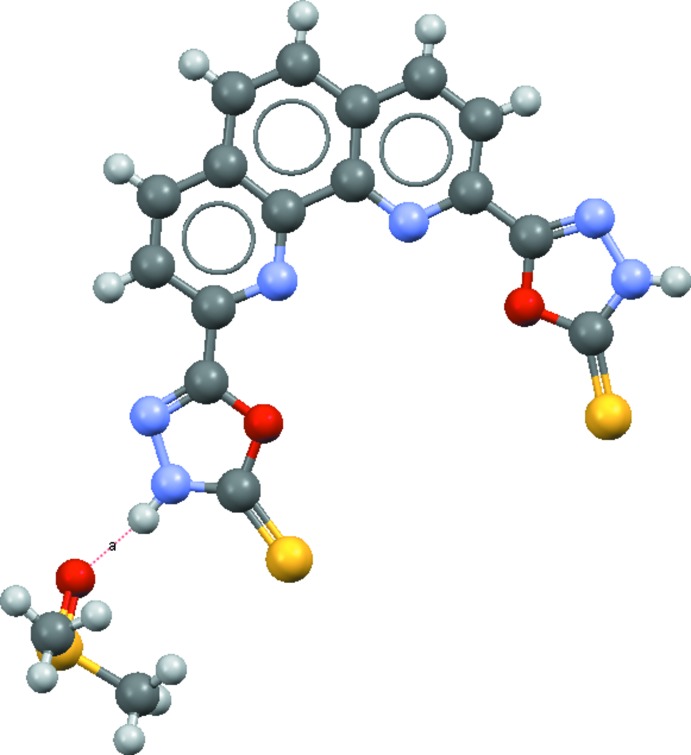
Hydrogen bonding interaction with a DMSO solvent molecule

**Fig. 3.**
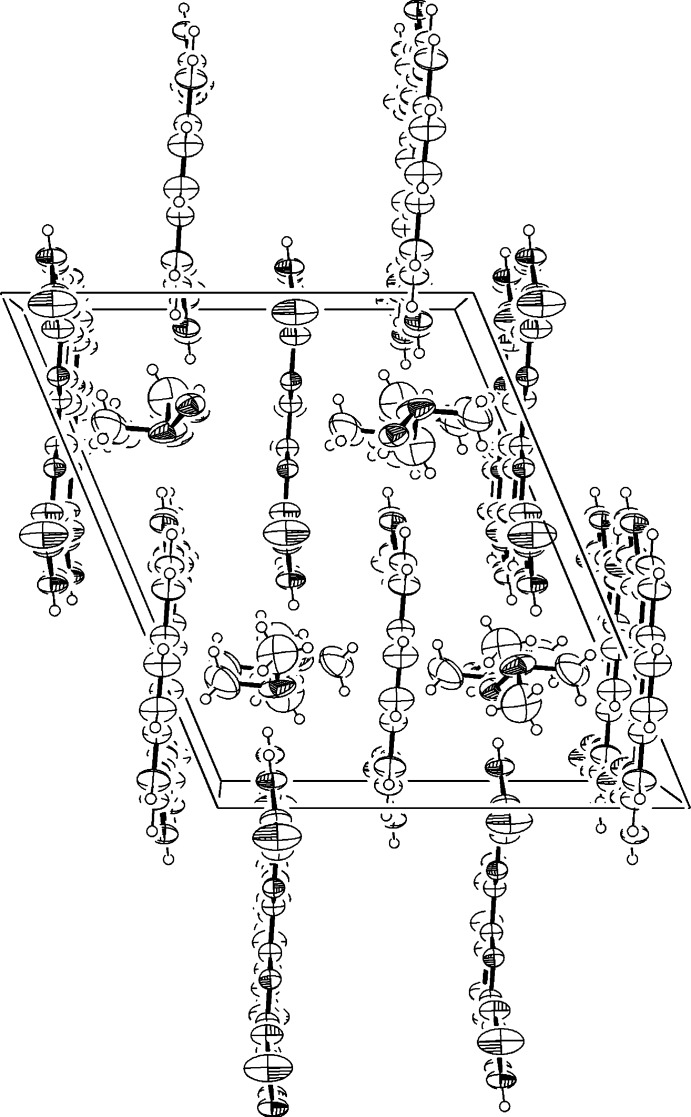
Packing diagram viewed along the normal to (101). Note the solvent molecules are positioned between two compounds making hydrogen bonding feasible

## supplementary crystallographic information

### 1. Comment

Compounds containing the oxadiazol moieties are known for their antimicrobial and anticancer activities. The title compound was prepared and characterized with the aim of synthesizing a series of compounds that are potentially active. These compounds were synthesized by a new process.

The title compound crystallized as a dimethyl sulfoxide (DMSO) solvate. The O atoms of the solvent form strong hydrogen bonds with the N—H centers of the diazole moiety.

The molecules are essentially planar, although the plane of the oxadiazolic five membered thionyl groups are slightly rotated from the phenanthrolene plane (the two least-squares planes form an angle of 8.86 (5)°). The molecules are arranged in planar sheets, with each pair of thiol S atoms pointing at the two apical hydrogen atoms on the adjacent phenanthroline groups.

### 2. Experimental

1,10-Phenanthroline-2,9-Di-*S*-methylhydrazinecarbodithioate (0.100 g, 0.21 mmol) was dissolved in THF (30 mL) with heat until a clear solution was formed. Then a solution of ZnCl_2_ (0.015 g, 0.11 mmol) in THF (5 mL) was added drop-wise to the carbodithioate solution. The resulting mixture was refluxed for 2–4 hrs. After completion of the reaction, as indicated by TLC, the reaction mixture was allowed to cool to room temperature. The solvent was evaporated under reduced pressure. The product was washed with ether and dried under vacuum. Recrystallization from DMSO yielded white crystals suitable for diffraction (0.076 g, 95%Y). ^1^HNMR (DMSO-d6, p.p.m.): δH 8.8 (d, 2H), 8.4(d, 2H), 8.2 (s, 2H). ^13^CNMR (DMSO-d6, p.p.m.): δC 178, 159, 144, 141, 138, 130, 128, 122. IR ν (cm^-1^): 3230 (N—H), 1196 (C—O), 3100–3000(C—H), 1600–1500 (aromatic C=C), 1373 (C=S).

### 3. Refinement

One large residual peak near the DMSO solvent molecule appears to indicate an alternate position of the S atom (an inversion of the DMSO pyramid). The disorder of the S atom was refined to an occupancy of less than 5% for the minor position; the minor occupancy of the lighter atoms of the solvent molecule were not included in the model.

Non-hydrogen atoms were refined with anisotropic thermal parameters, and hydrogen atoms were included in calculated positions (riding model) with *U_iso_* set to 1.2 times the *U_eq_* of the parent atom. Refinement on F2 by full-matrix least-squares resulted in R1 = 0.0727 and wR2 = 0.1729 for 2742 reflections with *I* > *2σ (I)*.

### Crystal data

**Table:**

C_16_H_8_N_6_O_2_S_2_·2C_2_H_6_OS	*F*(000) = 1112
*M**_r_* = 536.66	*D*_x_ = 1.47 Mg m^−^^3^
Monoclinic, *C*2/*c*	Mo *K*α radiation, λ = 0.71075 Å
Hall symbol: -C 2yc	Cell parameters from 2562 reflections
*a* = 14.113 (11) Å	θ = 3–27.7°
*b* = 11.161 (8) Å	µ = 0.43 mm^−^^1^
*c* = 16.708 (12) Å	*T* = 277 K
β = 112.837 (14)°	Prism, white
*V* = 2425 (3) Å^3^	0.42 × 0.26 × 0.15 mm
*Z* = 4	

### Data collection

**Table:**

Rigaku XtaLAB mini diffractometer	1691 reflections with *I* > 2σ(*I*)
Graphite monochromator	*R*_int_ = 0.039
profile data from ω–scans	θ_max_ = 27.5°, θ_min_ = 3.0°
Absorption correction: multi-scan (*CrystalClear*; Pflugrath, 1999)	*h* = −18→16
*T*_min_ = 0.840, *T*_max_ = 0.938	*k* = −10→14
5592 measured reflections	*l* = −21→17
2741 independent reflections	

### Refinement

**Table:**

Refinement on *F*^2^	Primary atom site location: structure-invariant direct methods
Least-squares matrix: full	Secondary atom site location: difference Fourier map
*R*[*F*^2^ > 2σ(*F*^2^)] = 0.067	Hydrogen site location: inferred from neighbouring sites
*wR*(*F*^2^) = 0.171	H atoms treated by a mixture of independent and constrained refinement
*S* = 1.03	*w* = 1/[σ^2^(*F*_o_^2^) + (0.0524*P*)^2^ + 6.1166*P*] where *P* = (*F*_o_^2^ + 2*F*_c_^2^)/3
2741 reflections	(Δ/σ)_max_ = 0.001
164 parameters	Δρ_max_ = 0.30 e Å^−^^3^
0 restraints	Δρ_min_ = −0.59 e Å^−^^3^

### Special details

**Table:**

Geometry. All s.u.'s (except the s.u. in the dihedral angle between two l.s. planes) are estimated using the full covariance matrix. The cell s.u.'s are taken into account individually in the estimation of s.u.'s in distances, angles and torsion angles; correlations between s.u.'s in cell parameters are only used when they are defined by crystal symmetry. An approximate (isotropic) treatment of cell s.u.'s is used for estimating s.u.'s involving l.s. planes.
Refinement. Refinement of *F*^2^ against ALL reflections. The weighted *R*-factor *wR* and goodness of fit *S* are based on *F*^2^, conventional *R*-factors *R* are based on *F*, with *F* set to zero for negative *F*^2^. The threshold expression of *F*^2^ > 2σ(*F*^2^) is used only for calculating *R*-factors(gt) *etc*. and is not relevant to the choice of reflections for refinement. *R*-factors based on *F*^2^ are statistically about twice as large as those based on *F*, and *R*- factors based on ALL data will be even larger.

### Fractional atomic coordinates and isotropic or equivalent isotropic displacement parameters (Å^2^)

**Table:**

	*x*	*y*	*z*	*U*_iso_*/*U*_eq_	Occ. (<1)
S2	0.26830 (13)	1.33767 (13)	0.77108 (7)	0.0922 (6)	0.953 (3)
S2B	0.265 (2)	1.355 (3)	0.695 (2)	0.092*	0.047 (3)
O2	0.1971 (2)	1.2428 (2)	0.72136 (16)	0.0647 (8)	
C9	0.3844 (4)	1.3093 (7)	0.7638 (4)	0.140 (3)	
H9A	0.4190	1.2459	0.8031	0.211*	
H9B	0.4260	1.3803	0.7787	0.211*	
H9C	0.3730	1.2858	0.7055	0.211*	
C10	0.2382 (6)	1.4660 (5)	0.7056 (4)	0.130 (2)	
H10A	0.2309	1.4449	0.6478	0.195*	
H10B	0.2922	1.5238	0.7291	0.195*	
H10C	0.1748	1.4994	0.7041	0.195*	
S1	0.10833 (14)	1.36039 (10)	0.46674 (8)	0.1039 (6)	
O1	0.0925 (2)	1.1303 (2)	0.42720 (15)	0.0574 (7)	
N2	0.1421 (3)	1.1611 (3)	0.56291 (19)	0.0557 (8)	
N1	0.1384 (2)	1.0401 (2)	0.55250 (17)	0.0506 (7)	
N3	0.0448 (2)	0.9170 (2)	0.33922 (16)	0.0397 (6)	
C1	0.1091 (3)	1.0258 (3)	0.4711 (2)	0.0444 (8)	
C6	0.0494 (3)	0.7019 (3)	0.3399 (2)	0.0520 (9)	
C4	0.1181 (3)	0.8082 (3)	0.4726 (2)	0.0548 (9)	
H4	0.1506	0.8106	0.5328	0.066*	
C7	0.0238 (4)	0.5931 (3)	0.2927 (2)	0.0690 (12)	
H7	0.0409	0.5206	0.3223	0.083*	
C5	0.0973 (3)	0.7028 (3)	0.4299 (2)	0.0614 (11)	
H5	0.1148	0.6311	0.4606	0.074*	
C2	0.1163 (3)	1.2176 (3)	0.4891 (2)	0.0598 (10)	
C8	0.0250 (3)	0.8122 (3)	0.2969 (2)	0.0425 (7)	
C3	0.0899 (3)	0.9131 (3)	0.4245 (2)	0.0439 (8)	
H2	0.164 (3)	1.195 (3)	0.616 (3)	0.062 (11)*	

### Atomic displacement parameters (Å^2^)

**Table:**

	*U*^11^	*U*^22^	*U*^33^	*U*^12^	*U*^13^	*U*^23^
S2	0.1324 (13)	0.1011 (10)	0.0456 (6)	−0.0709 (9)	0.0373 (7)	−0.0308 (6)
O2	0.0782 (19)	0.0651 (16)	0.0492 (14)	−0.0269 (14)	0.0228 (13)	−0.0169 (12)
C9	0.090 (4)	0.188 (7)	0.113 (5)	−0.065 (5)	0.007 (4)	0.027 (5)
C10	0.185 (7)	0.066 (3)	0.157 (6)	−0.052 (4)	0.085 (5)	−0.023 (4)
S1	0.1906 (17)	0.0392 (6)	0.0635 (7)	−0.0070 (7)	0.0292 (9)	0.0048 (5)
O1	0.0884 (19)	0.0388 (13)	0.0382 (13)	−0.0047 (12)	0.0170 (13)	0.0007 (10)
N2	0.081 (2)	0.0418 (16)	0.0369 (16)	−0.0088 (15)	0.0144 (15)	−0.0045 (13)
N1	0.069 (2)	0.0379 (14)	0.0381 (15)	−0.0046 (14)	0.0135 (14)	0.0010 (11)
N3	0.0452 (16)	0.0351 (14)	0.0379 (14)	0.0002 (11)	0.0151 (12)	0.0014 (10)
C1	0.052 (2)	0.0377 (17)	0.0382 (18)	−0.0035 (15)	0.0123 (15)	0.0050 (13)
C6	0.069 (2)	0.0350 (17)	0.0465 (19)	0.0037 (16)	0.0168 (17)	0.0037 (14)
C4	0.069 (3)	0.050 (2)	0.0354 (18)	0.0045 (18)	0.0087 (17)	0.0039 (15)
C7	0.109 (4)	0.0306 (17)	0.058 (2)	0.004 (2)	0.022 (2)	0.0056 (15)
C5	0.088 (3)	0.0379 (19)	0.047 (2)	0.0087 (19)	0.014 (2)	0.0112 (15)
C2	0.085 (3)	0.044 (2)	0.044 (2)	−0.0051 (19)	0.0176 (19)	−0.0021 (15)
C8	0.051 (2)	0.0338 (16)	0.0416 (17)	0.0002 (14)	0.0163 (15)	0.0008 (13)
C3	0.053 (2)	0.0374 (17)	0.0399 (17)	−0.0011 (15)	0.0162 (16)	0.0014 (13)

### Geometric parameters (Å, º)

**Table:**

S2—O2	1.474 (3)	N1—C1	1.269 (4)
S2—C9	1.719 (7)	N3—C3	1.317 (4)
S2—C10	1.751 (6)	N3—C8	1.338 (4)
C9—H9A	0.9600	C1—C3	1.449 (4)
C9—H9B	0.9600	C6—C5	1.389 (5)
C9—H9C	0.9600	C6—C8	1.400 (4)
C10—H10A	0.9600	C6—C7	1.416 (5)
C10—H10B	0.9600	C4—C5	1.348 (5)
C10—H10C	0.9600	C4—C3	1.387 (5)
S1—C2	1.631 (4)	C4—H4	0.9300
O1—C1	1.348 (4)	C7—C7^i^	1.321 (8)
O1—C2	1.365 (4)	C7—H7	0.9300
N2—C2	1.305 (5)	C5—H5	0.9300
N2—N1	1.360 (4)	C8—C8^i^	1.448 (6)
N2—H2	0.89 (4)		
			
O2—S2—C9	106.7 (3)	O1—C1—C3	120.2 (3)
O2—S2—C10	106.7 (3)	C5—C6—C8	118.0 (3)
C9—S2—C10	96.5 (3)	C5—C6—C7	121.4 (3)
S2—C9—H9A	109.5	C8—C6—C7	120.6 (3)
S2—C9—H9B	109.5	C5—C4—C3	118.4 (3)
H9A—C9—H9B	109.5	C5—C4—H4	120.8
S2—C9—H9C	109.5	C3—C4—H4	120.8
H9A—C9—H9C	109.5	C7^i^—C7—C6	121.0 (2)
H9B—C9—H9C	109.5	C7^i^—C7—H7	119.5
S2—C10—H10A	109.5	C6—C7—H7	119.5
S2—C10—H10B	109.5	C4—C5—C6	119.6 (3)
H10A—C10—H10B	109.5	C4—C5—H5	120.2
S2—C10—H10C	109.5	C6—C5—H5	120.2
H10A—C10—H10C	109.5	N2—C2—O1	105.5 (3)
H10B—C10—H10C	109.5	N2—C2—S1	131.2 (3)
C1—O1—C2	105.4 (3)	O1—C2—S1	123.3 (3)
C2—N2—N1	112.1 (3)	N3—C8—C6	122.5 (3)
C2—N2—H2	126 (2)	N3—C8—C8^i^	119.07 (17)
N1—N2—H2	122 (2)	C6—C8—C8^i^	118.39 (19)
C1—N1—N2	104.0 (3)	N3—C3—C4	124.3 (3)
C3—N3—C8	117.2 (3)	N3—C3—C1	117.6 (3)
N1—C1—O1	112.9 (3)	C4—C3—C1	118.1 (3)
N1—C1—C3	126.8 (3)		
			
C2—N2—N1—C1	−0.5 (5)	C3—N3—C8—C6	0.7 (5)
N2—N1—C1—O1	−0.5 (4)	C3—N3—C8—C8^i^	179.7 (4)
N2—N1—C1—C3	−178.3 (3)	C5—C6—C8—N3	−0.9 (6)
C2—O1—C1—N1	1.3 (4)	C7—C6—C8—N3	177.8 (4)
C2—O1—C1—C3	179.2 (3)	C5—C6—C8—C8^i^	−180.0 (4)
C5—C6—C7—C7^i^	178.4 (6)	C7—C6—C8—C8^i^	−1.3 (6)
C8—C6—C7—C7^i^	−0.2 (8)	C8—N3—C3—C4	0.2 (5)
C3—C4—C5—C6	0.5 (6)	C8—N3—C3—C1	−178.3 (3)
C8—C6—C5—C4	0.3 (6)	C5—C4—C3—N3	−0.8 (6)
C7—C6—C5—C4	−178.4 (4)	C5—C4—C3—C1	177.7 (4)
N1—N2—C2—O1	1.2 (5)	N1—C1—C3—N3	169.7 (4)
N1—N2—C2—S1	179.8 (4)	O1—C1—C3—N3	−7.9 (5)
C1—O1—C2—N2	−1.5 (4)	N1—C1—C3—C4	−9.0 (6)
C1—O1—C2—S1	179.8 (3)	O1—C1—C3—C4	173.4 (3)

Symmetry code: (i) −*x*, *y*, −*z*+1/2.

### Hydrogen-bond geometry (Å, º)

**Table:**

*D*—H···*A*	*D*—H	H···*A*	*D*···*A*	*D*—H···*A*
N2—H2···O2	0.89 (4)	1.73 (4)	2.617 (4)	172 (4)
C9—H9*C*···O1^ii^	0.96	2.62	3.399 (7)	138
C10—H10*B*···O2^iii^	0.96	2.57	3.317 (6)	135
N2—H2···S2*B*	0.89 (4)	2.36 (5)	3.10 (3)	140 (3)

Symmetry codes: (ii) −*x*+1/2, −*y*+5/2, −*z*+1; (iii) −*x*+1/2, *y*+1/2, −*z*+3/2.
